# Monosodium iodoacetate-induced subchondral bone microstructure and inflammatory changes in an animal model of osteoarthritis

**DOI:** 10.1515/biol-2022-0079

**Published:** 2022-07-16

**Authors:** Zheming Bao, Mengli Chen, Chen Li, Qing Shan, Yichen Wang, Wenshan Yang

**Affiliations:** Department of Pharmacy, Medical Supplies Centre of PLA General Hospital, No. 28, Fuxing Road, Haidian District, Beijing 100853, China; Orthopedics Department, 960th Hospital of PLA Joint Service Support Force, Jinan, China

**Keywords:** animal model, osteoarthritis, bone microarchitecture, iodoacetic acid

## Abstract

The monosodium iodoacetate (MIA)-induced osteoarthritis (OA) may lead to cartilage degeneration and histopathological lesions. However, the correlation between inflammatory reaction and subchondral bone remodeling in a rodent osteoarthritic model is ambiguous. In this study, intra-articular injection of MIA was performed in 36 four-week-old specific pathogen-free male Wistar rats to induce OA. After 4 weeks of intervention, changes in intrinsic structural properties of the subchondral bones were measured, and the histological evaluation, as well as biochemical analysis, was conducted. We found that intra-articular injection of MIA increased chondrocyte apoptosis and promoted cartilage matrix degradation, such as cartilage surface defects and shallow or disappearing staining. MIA also induced inflammation, improved the expression of IL-1β, TNF-α, and matrix metalloproteinase, and decreased the expression of cartilage-specific proteins with the extension of modeling time. Meanwhile, the MIA also significantly accelerated the subchondral bone remodeling, as shown by the decreased subchondral bone density, thinning of trabeculae, disordered cartilage structure, and morphology. In conclusion, we have shown that MIA-induced rodent osteoarthritic model would cause decreased subchondral bone density, sparse trabecular bone, and other manifestations of osteoporosis accompanied by an inflammatory response, which would worsen with the progression of modeling time. Our results suggest that different phases of MIA-induced OA are associated with the changes in subchondral bone microstructure and the progression of local inflammation.

## 1 Introduction

Osteoarthritis (OA), a degenerative joint condition, is characterized by progressive degeneration of articular cartilage, subchondral bone remodeling, inflammation, and pain [1]. OA is especially initiated by excessive mechanical loading on the weight-bearing joints [2], leading to functional disability, breakdown of the articular cartilage, and economic stress for both the individual and government [3].

Proinflammatory cytokines and inflammatory mediators are known to play an important role in the pathogenesis of OA [4]. A number of studies have demonstrated that inflammatory cytokine levels are elevated in OA patients [5]. Interleukin-1 beta (IL-1β) and tumor necrosis factor-alpha (TNF-α), as the principal proinflammatory mediators, induce the production of matrix metalloproteinases (MMPs), which may lead to the downregulation of collagen-II and aggrecan in articular cartilage [6,7]. OA tends to deteriorate when the self-perpetuating inflammation and cartilage wear overwhelm the reparative capacity of the chondrocytes [7,8].

On the other hand, compensatory changes in the subchondral bone structure are often neglected. Typically, most OA studies focus on the changes in articular cartilage. There is still abundant data indicating an important role of the subchondral bone in the pathogenesis of OA [9,10]. Osteoclastic activity and bone resorption of subchondral bone are increased in the early stage of OA [2]. Furthermore, the anabolic and catabolic processes of bone tissue are manifested unbalanced in osteoarthritic subchondral bone [11] while producing various cytokines that seep through the bone–cartilage interface and initiate chondrocyte hypertrophy and promote cartilage degradation [12]. All in all, the osteoarthritic subchondral bone would influence the integrity of the overlying cartilage biologically and mechanically [13].

*In vivo* osteoarthritic rat model is commonly induced by monosodium iodoacetate (MIA), a glyceraldehyde-3-phosphate dehydrogenase inhibitor that simulates the phenomena observed in human OA such as inflammatory response [5,14], progressive loss of chondrocytes, and lesions in the cartilage [1,3,15,16]. However, numerous studies have used MIA as an inducer in animal models of knee OA [5,17,18]. It is unclear whether the severity of articular cartilage degeneration induced by MIA is related to the expression of intraarticular inflammatory factors.

As local inflammation plays an important role in the pathogenesis of OA, we hypothesized that different phases of MIA-induced OA may be associated with the changes in subchondral bone microstructure and the progression of local inflammation. Therefore, the present study intended to investigate the expression of inflammation-associated cytokines and characteristics of articular cartilage in the joints of the osteoarthritic rat model induced by MIA. In the meantime, histomorphological bone remodeling in the subchondral bone microstructure during the progression of OA was observed *in vivo*.

## 2 Materials and methods

### 2.1 Animals

This animal study is subject to and includes all ARRIVE guidelines. For this experiment, 36 four-week-old specific pathogen-free male Wistar rats weighing 240–260 g were selected. The rats were kept in plastic cages at room temperature (21–24°C) in a 12 h light and dark cycle with free access to food and water. All experimental animals were fed in the laboratory animal center.

The rats were randomly and equally divided into three groups: (a) sham group (*n* = 12) was given sterile normal saline for 4 weeks; (b) MIA two-week group (*n* = 12) was received MIA for 2 weeks; and (c) MIA four-week group (*n* = 12) was received MIA for 4 weeks.


**Ethical approval:** The research related to animal use has complied with all the relevant national regulations and institutional policies for the care and use of animals and was approved by the Animal Ethics Committee of PLA General Hospital (Approval number: SQ2020127).

### 2.2 Induction of knee OA

Rats in all groups were anesthetized by intraperitoneal injection of 3% pentobarbital sodium (45 mg/kg) and injected with a 50 μL solution into the right knee joint using a microsyringe (26 G). Rats in the MIA group were intra-articular injected with 50 µL of 3 mg MIA [18,19], which were diluted in sterile normal saline. On the day of injection, the solution of MIA, which was purchased from Aladdin (Shanghai, China), was freshly prepared in sterile normal saline at 60 g/L concentration. The animals of the sham group received an intra-articular injection of an equivalent volume of saline.

The limbs of the rats were gently strapped with hemp ropes on an operating table in a supine position. The knee joints of the rats were bent at a 90° angle so that the patellar ligaments were positioned, and the needles passed through the parapatellar joint space. In the sham group, the same surgical procedure was performed.

After 1-week adaptive breeding, the sham and MIA 4-week groups were injected in the second week for 4 weeks. The MIA 2-week group adapted the treatment in the fourth week for 2 weeks.

Rats were sacrificed by pentobarbital overdose after 4 weeks following adaptive breeding. The distal femur knee joint specimens were isolated and stored in liquid nitrogen for further experiments, while proximal tibia samples were harvested for histological assessment.

### 2.3 Histological analysis

We assessed the effect of MIA on cartilage degeneration of knee joints in OA rats by histological observation. The right knees of the rats were dissected, and the tibial-femoral joints were removed with bone scissors without soft connective tissues. The proximal tibias were fixed in 4% paraformaldehyde for 48 h and then transferred to 10% ethylenediaminetetraacetic acid for decalcification, with the solution being replaced every 5 days for 4 weeks [18]. Afterward, the paraffin blocks of joints were embedded and sectioned on a coronal plane at the thickness of 5 µm using a histotome (Leica RM2016, Shanghai, China). These samples were subsequently processed for histopathology and stained with haematoxylin–eosin (H&E), safranin fast green, and toluidine blue. Tibial cartilages and subchondral bones below the articular cartilage were evaluated using an OOCHAS (OA Cartilage Histopathology Assessment System) score method [20]. Histological evaluation was performed by a blinded pathologist. Joint evaluation aspects included OA depth progression into cartilage and joint involvement. The semi-quantitative method was used to produce an OA score, an index of combined grade, and a stage with a range of 0–24 [20].

All slides were observed and analyzed using a light microscope (Nikon Eclipse Ti2, Tokyo, Japan) equipped with imaging software (NIS elements, Tokyo, Japan). At least three slides were evaluated for each tissue sample.

### 2.4 Micro-computed tomography (micro-CT) scanning

The microarchitectural deterioration in MIA-induced OA models was conducted using micro-CT (Quantum GX, PerkinElmer, Kansas, USA). The formalin-fixed tibial bones were placed vertically and flat in the sample tank so that the longitudinal axis of the specimen was perpendicular to the ray. The scanning voltage was set at 70 kV and the current at 114 µA. The field of volume was obtained at 25 mm with voxel dimensions of 50 μm. All specimens were exposed for 10 min under the CT scanner. Reconstructions of the micro-CT image slices were performed with Mimics Research 21.0 software.

#### 2.4.1 Histomorphometric analysis of trabecular bone

A total of 50 layers of scanning images were selected as the Region of Interest to measure the microstructural trabecular parameters, including bone mineral density (BMD, mg/cc), bone volume fraction (BV/TV, %), connectivity density (Conn.D, mm^−3^), trabecular thickness (Tb.Th, mm), and trabecular separation (Tb.Sp, mm).

The subchondral trabecular region started below the subchondral plate and extended distally toward the metaphyseal trabecular, excluding both the cortical bone and growth plate interface. Subchondral bone parameters were analyzed by Analyze 12.0 software.

### 2.5 Quantitative real-time polymerase chain reaction analysis (QRT-PCR)

Approximately 20 mg of the distal femur tissues containing surface cartilage was disrupted in liquid nitrogen utilizing mortar and pestle, and total RNA was isolated by Trizol (Beyotime, Shanghai, China) using the protocol in the manual. The RNA yield was determined by measuring the absorbance at 260 nm, and purity was assessed according to the ratio of absorbance readings at 260–280 nm using the NanoPhotometer NP80 (Implen GmbH, München, Germany). The RT First Strand cDNA Synthesis Kit (Servicebio, Wuhan, China) was used to generate complementary DNA (cDNA) using 1 μg of total RNA. QRT-PCR was performed in a BIO-RAD CFX96 Real-Time PCR System (BIO-RAD, California, USA) using the SYBR Green qPCR Master Mix (Servicebio, Wuhan, China). β-Actin was used as the housekeeping gene, and the fold changes in relative gene expression were calculated using the 2^−ΔΔCt^ method. Sequence-specific primers for cDNA amplified are listed in Table A1.

### 2.6 Western blot analysis

Samples were cut into small fragments, and the tissue sample is ground into a homogenate and lysed by RIPA lysis buffer (Beyotime, Shanghai, China) with 1 mM PMSF. The total protein concentration of each sample was measured using a BCA Protein Assay Kit (Beyotime, Shanghai, China), and the protein concentration of each sample was balanced and denatured by a metal bath; 20 μg of protein lysate was loaded on 8–12% sodium dodecyl sulfate–polyacrylamide gel electrophoresis (SDS-PAGE) in tris-glycine-SDS buffer (2 h, 100 V), followed by transfer to a polyvinylidene difluoride (PVDF) membrane for 1.5 h at 300 mA. Afterward, the PVDF membrane blocking was done for 10 min by QuickBlock™ Blocking Buffer (Beyotime, Shanghai, China) at room temperature and then incubated with primary antibodies in QuickBlock™ Primary Antibody Dilution Buffer (Beyotime, Shanghai, China) against MMP13 (1:1,000, Beyotime, AF7479), TNF-α (1:1,000, Beyotime, AF8208), and β-actin (1:2,000, Abmart, P30002) at 4°C overnight. After washing three times in tris-buffered saline tween, the membranes were incubated for 1 h at room temperature with a horseradish peroxidase-conjugated secondary antibody (1:1,000, Beyotime, A0208). Finally, bands were visualized using enhanced chemiluminescence system G: BOX Chemi XRQ (Syngene, Cambridge, UK) the next day. Using an imaging analysis system, the signal from positive bands was calculated relative to the signal from the internal control (β-actin-positive bands).

### 2.7 IL-1β Immunofluorescence

Three sections from each knee were selected for IL-1β immunofluorescence. The deparaffinized and rehydrated sections were incubated with ethylene diamine tetraacetic acid (PH8.0) antigen retrieval buffer in a microwave oven and kept warm for 15 min. After blocking with 3% bovine serum albumin, the sections were incubated with rabbit anti-rat IL-1β (1:100, Beyotime, AF7209) overnight at 4°C. Next, the sections were incubated with Cy3-conjugated Goat anti-rabbit IgG (1:200, Servicebio, GB21303) at room temperature for 50 min. Finally, after the diamidinyl phenyl indole counterstain in the nucleus, the sections were incubated with spontaneous fluorescence quenching reagent for about 5 min. All images were acquired at 400× magnification using a fluorescence microscope (Nikon E100, Tokyo, Japan) with Nikon DS-U3 software (Nikon, Tokyo, Japan).

### 2.8 Statistical analyses

Western blot bands were quantitatively analyzed with Image J software (version 1.52, Wayne Rasband, USA). The data were analyzed using GraphPad Prism 8.0 software (San Diego, USA). The Shapiro–Wilk test was used to evaluate the normality of the data distribution. The results were presented as the means ± standard deviation. Kruskal–Wallis nonparametric analyses were used to compare cartilage degeneration scores, followed by the Dunn post hoc test. One-way analysis of variance followed by the least significant difference *post hoc* test was used to determine statistical differences. *P* values less than 0.05 were considered statistically significant.

## 3 Results

### 3.1 Histopathology in MIA-induced OA rats

**Figure 1:**
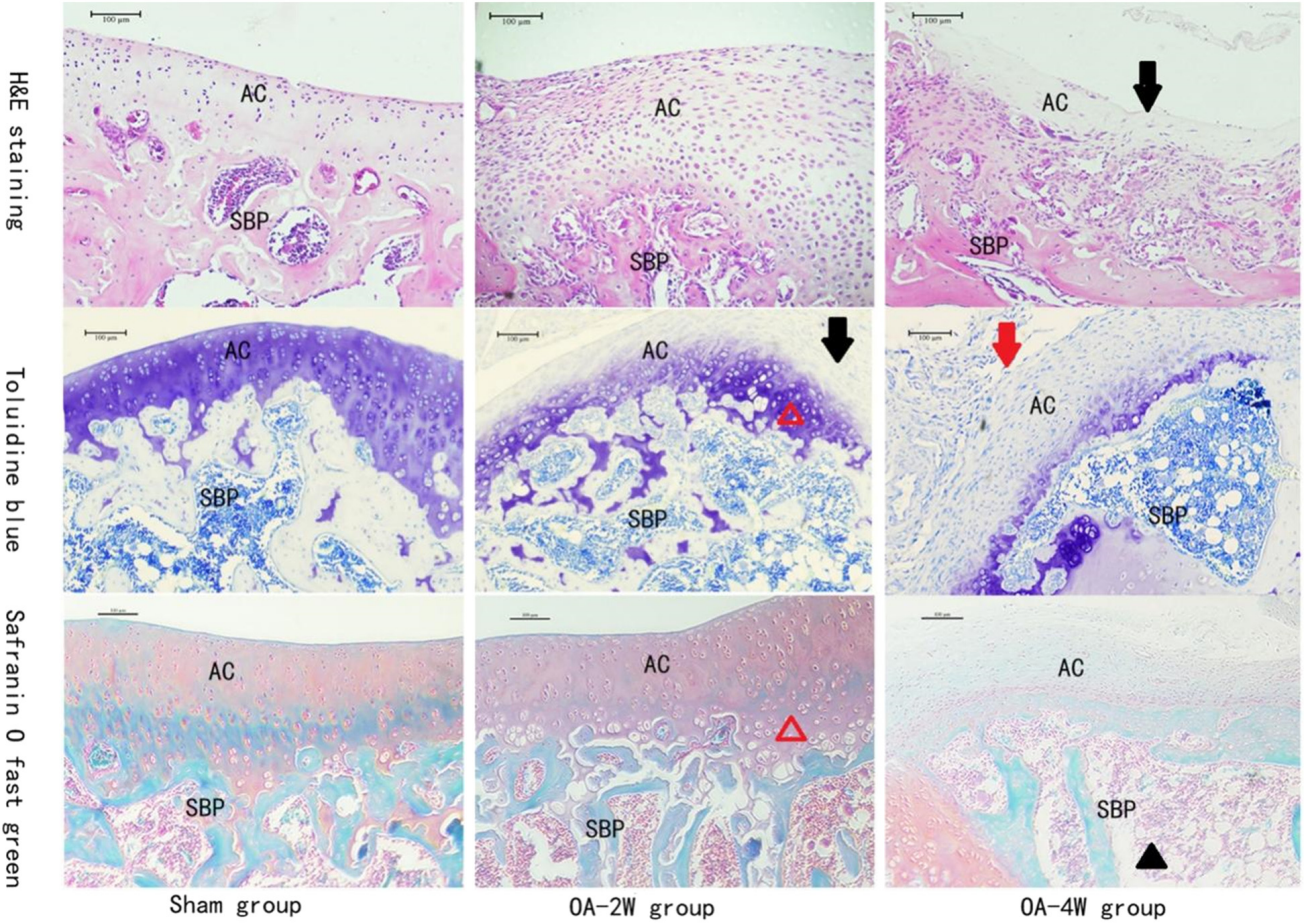
Representative micrographs of rat tibial joints receiving different treatments. Red arrow: a rough surface with fibrous degeneration; black arrow: absence of staining in cartilage surface; black triangle: increased trabecular space; red triangle: hypertrophy and vacuolated chondrocytes Abbreviation: SBP: subchondral bone plate; AC, articular cartilage. Sham group: animals received sterile saline 0.9% for 4 weeks; OA-2W group: animals received MIA for 2 weeks; OA-4W group: animals received MIA for 4 weeks. Scale bar, 100 μm.

To understand the articular cartilage changes after MIA modeling, the articulation of the animals that received MIA was investigated under histological observations at different modeling stages. The cartilage histology was evaluated in all groups on day 28, and representative micrographs are shown in Figure 1.

The saline-injected group did not show any remarkable lesions in the articular cartilage. The histological sections showed that the proximal tibias of the rats in the sham group were stained well, and the cartilage layers were integral. The knee joints of MIA rats were erosive compared to those of the sham group. MIA-injected animals showed erosion damage in the articular cartilage of the bilateral tibial plateau. Injuries were more severe in the four-week group, predominantly in the junction of the femoral condyles, due to the greater contact between the tibiofemoral structures. Simultaneously, a rough surface with fibrous degeneration was presented in a partial thinness lesion district of cartilage, with an extensive area covered with fibrous tissue. Moreover, accompanied by the absence of safranin O and toluidine blue staining, the animals injected with MIA underwent a mass of chondrocyte loss in the knee joint area compared with the animals in the sham group treated with normal saline, and the chondrocytes in the deep layer of articular cartilage had significant aggrecan loss and disappearance of chondrocytes.

**Figure 2:**
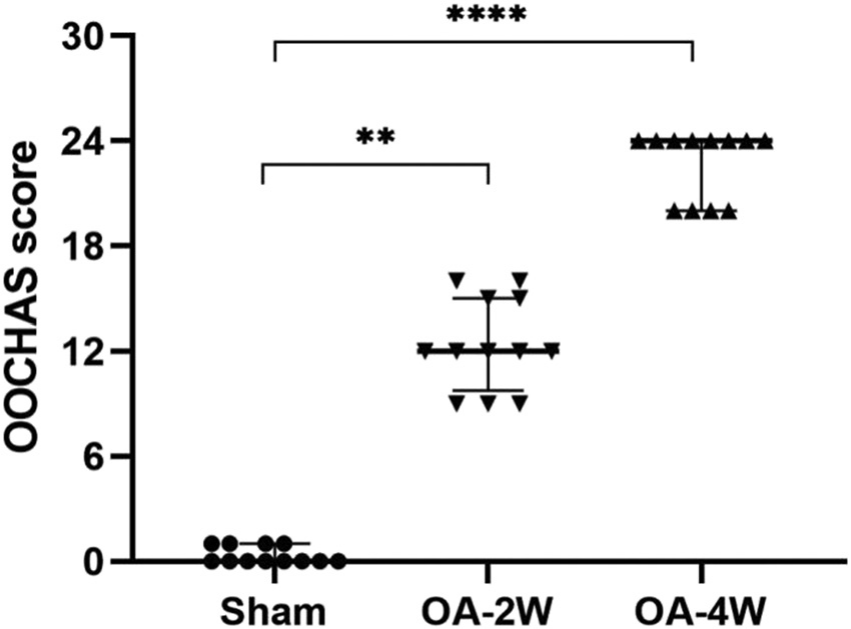
OOCHAS scores in the three groups. *n* = 12 per group. **P < 0.01 compared to the sham group and **** *P* < 0.0001 compared to the sham group. Sham: animals received sterile saline 0.9% for 4 weeks; OA-2W: animals received MIA for 2 weeks; OA-4W: animals received MIA for 4 weeks.

As shown in Figure 2, the OOCHAS scores of the MIA-injected group were significantly higher than those of the sham group, which was 0(0, 1). Simultaneously, treatment with MIA exacerbated the degenerative changes prominently as the duration of injection extended (*P* < 0.05). By week two, the MIA-induced joints exhibited moderate cartilage degeneration, and the OOCHAS score was 12(9.75, 15). By week four, severe cartilage degeneration occurred, and the OOCHAS score was 24 (20, 24).

### 3.2 micro-CT images

**Figure 3:**
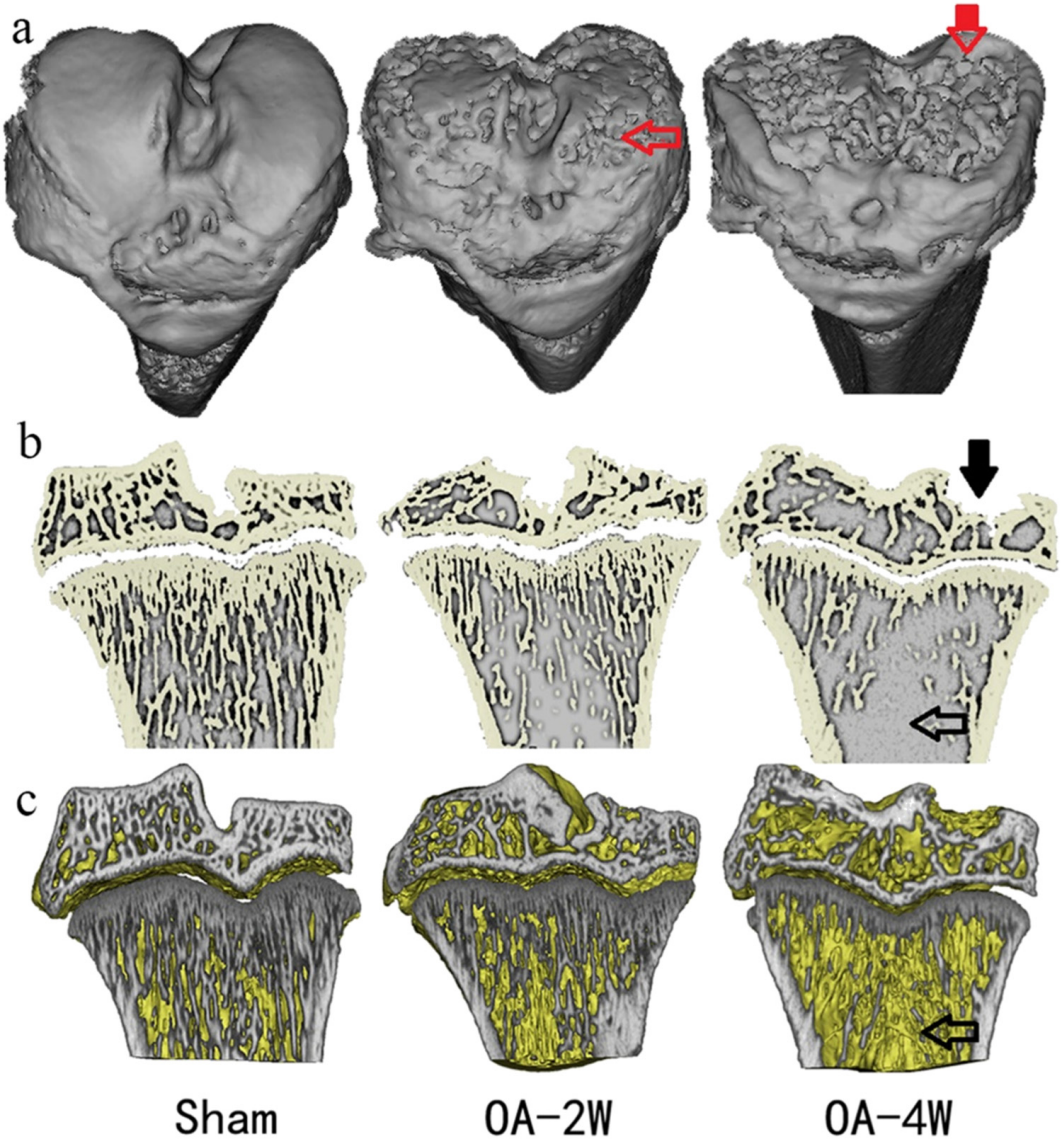
(a) Representative micro-CT three-dimensional images of rat tibial bone in three groups. (b) Representative micro-CT two-dimensional sectional view of the knee. (c) Representative micro-CT three-dimensional sectional view of the knee. Red arrow: The MIA groups exhibited a rough surface on the cartilage and an extensive area of cartilage defects. Black arrow: There was significant cartilage loss and trabeculae missing together with widening of Tb.Sp in the MIA-injected group. Sham: animals received sterile saline 0.9% for 4 weeks; OA-2W: animals received MIA for 2 weeks; OA-4W: animals received MIA for 4 weeks.

As shown in Figure 3a, micro-CT three-dimensional reconstructed images confirmed that the articular cartilage was normal in the sham group presented, and the articular surface was complete and smooth, while the articular cartilage was rough in the MIA-injected groups, with osteophyte formation. Micro-CT analysis of tibial bone by micro-CT indicated significant cartilage degeneration in MIA groups relative to the sham group. Moreover, the MIA groups showed an extensive articular cartilage collapse and an extensive cartilage defect in the tibial plateau with the extension of time. Furthermore, after MIA injection, micro-CT analysis showed a significant loss of subchondral bone in MIA-injected groups (Figure 3b and c). The subchondral bone collapse and cracks of bone trabeculae, as well as loss of reticular structure in the tibial plateau, were increased compared to the intact and regular trabecular network of the sham group. What’s more, thinning of the subchondral bone trabeculae was observed by micro-CT two-dimensional and three-dimensional sectional view, together with widening of Tb.Sp. Injection of MIA at the dose of 3 mg showed significant changes in the trabecular structure of subchondral bone, articular surface cartilage, as well as in BMD, which gradually aggravated degradation with the progression of modeling time.

### 3.3 Histomorphometric analysis of trabecular bone

**Figure 4:**
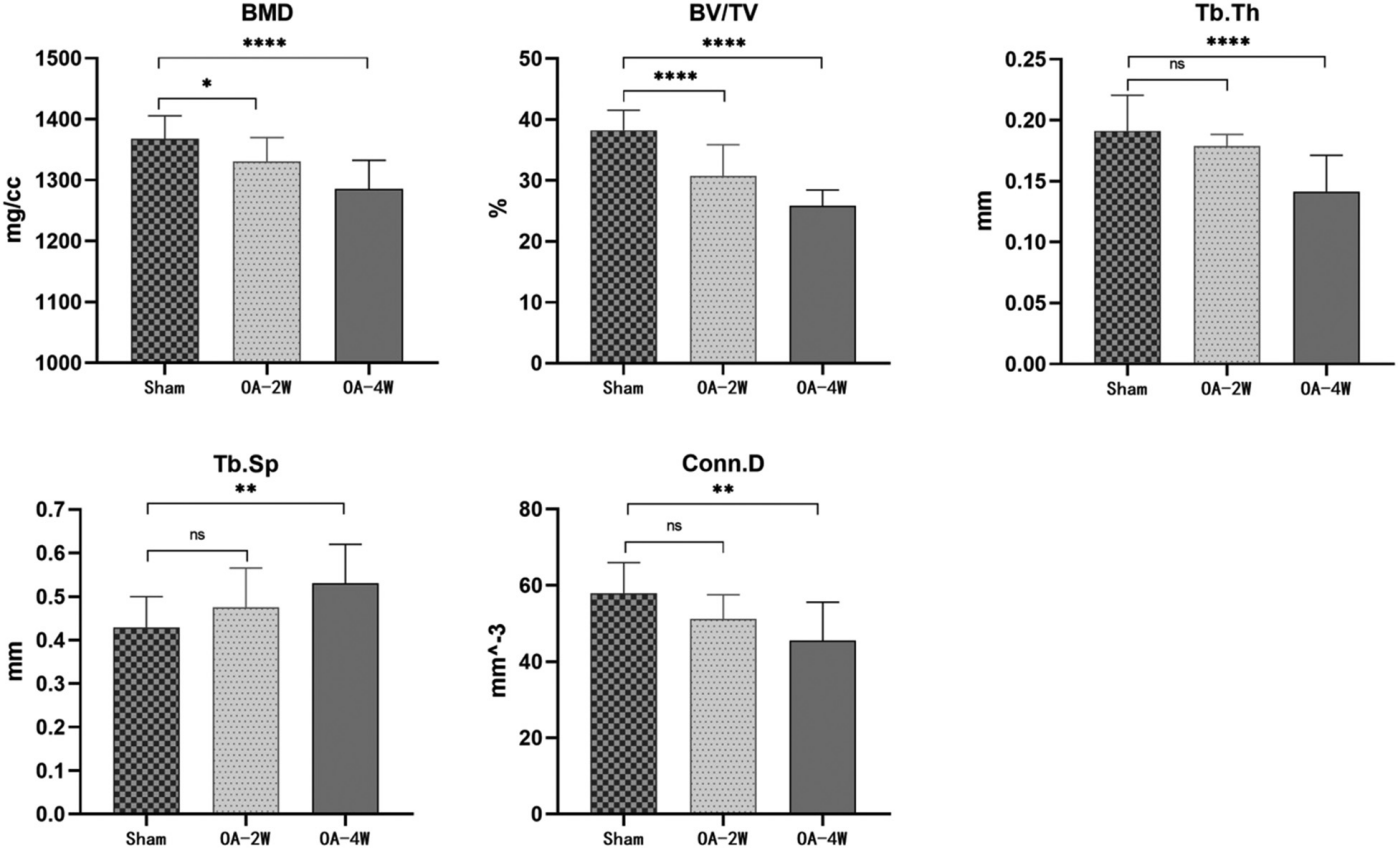
Quantitative micro-CT analysis of various parameters of tibial subchondral bone. BMD, BV/TV, Tb. Th, Tb. SP, and Conn. D. *n* = 9 per group. *P < 0.05 compared to the sham group, **P < 0.01 compared to the sham group, and **** *P* < 0.0001 compared to the sham group, ns stands for no statistical significance compared to the sham group. Sham: animals received sterile saline 0.9% for 4 weeks; OA-2W: animals received MIA for 2 weeks; OA-4W: animals received MIA for 4 weeks.

Intra-articular injection of MIA led to articular cartilage damage and increased bone remodeling as the disease progressed, followed by subchondral bone deterioration. BMD and Tb.Th were significantly declined in the subchondral bone of rat tibia treated with MIA at both weeks two and week four simultaneously, and aggravated osteoporosis was observed over time compared to the sham group (Figure 4). Micro-CT of the subchondral bone site showed that MIA-induced reduction of BV/TV to (30.72 ± 5.122)% in the MIA two-week group and (25.81 ± 2.608)% in the MIA four-week group compared to (38.22 ± 3.284)% in the sham group. Decrease in subchondral BV/TV after MIA-induced OA eventually led to the decline of BMD, which was (1330.66 ± 38.99) mg/cc in the MIA two-week group and (1285.5 ± 47.08) mg/cc in MIA four-week group compared to (1367.97 ± 37.43) mg/cc in the sham group (Figure 4). Tb.Th in the sham group was (0.1912 ± 0.0293) mm, decreased to (0.1750 ± 0.0194) mm in MIA two-week group, and decreased to (0.1414 ± 0.0299) mm in MIA four-week group (Figure 4). MIA significantly thinned bone trabeculae in subchondral bone leading to increased Tb.Sp in MIA groups, which was indicated by a significant increase to (0.4750 ± 0.0903) mm in MIA two-week group and (0.5307 ± 0.0893) mm in MIA four-week group compared to (0.4288 ± 0.0710) mm in the sham group (Figure 4). On the other hand, trabecular connectivity decreased from (57.94 ± 8.050) mm^−3^ in the sham group to (52.39 ± 8.781) mm^−3^ in MIA two-week group and to (45.48 ± 10.14) mm^−3^ in MIA four-week group (Figure 4).

### 3.4 Expression of inflammatory cytokines and cartilage-associated proteins

**Figure 5:**
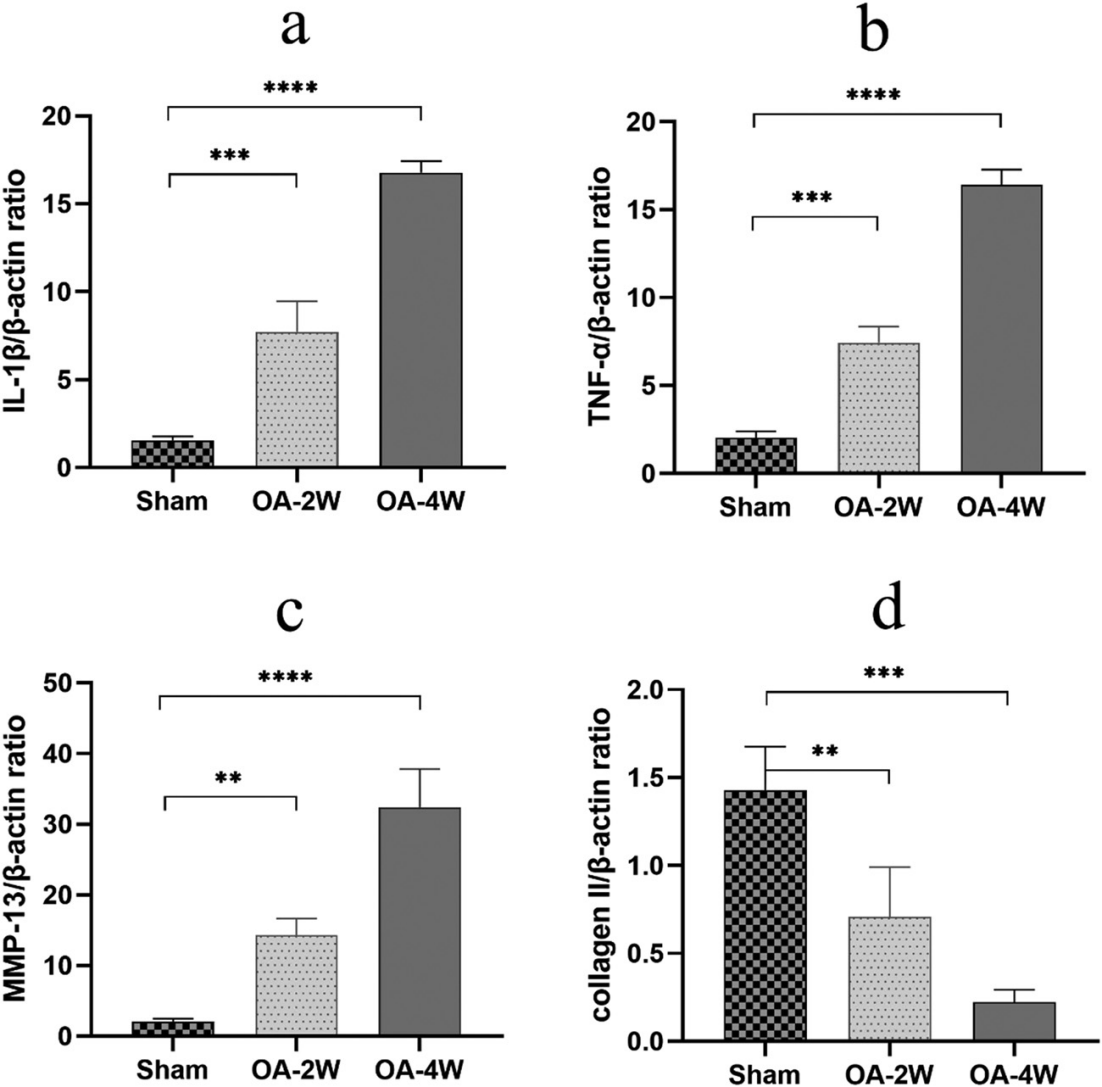
Relative mRNA expression of genes: (a) IL1-β, (b) TNF-α, (c) MMP-13, and (d) collagen II in distal femur containing cartilage and subchondral region. All values are expressed as Mean ± SD. (*n* = 9/group). **P < 0.01, ***P < 0.001, and ****P < 0.0001 compared to the sham group. IL-1β, TNF-α, Matrix metalloproteinase 13(MMP-13). Sham: animals received sterile saline 0.9% for 4 weeks; OA-2W: animals received MIA for 2 weeks; OA-4W: animals received MIA for 4 weeks.

**Figure 6:**
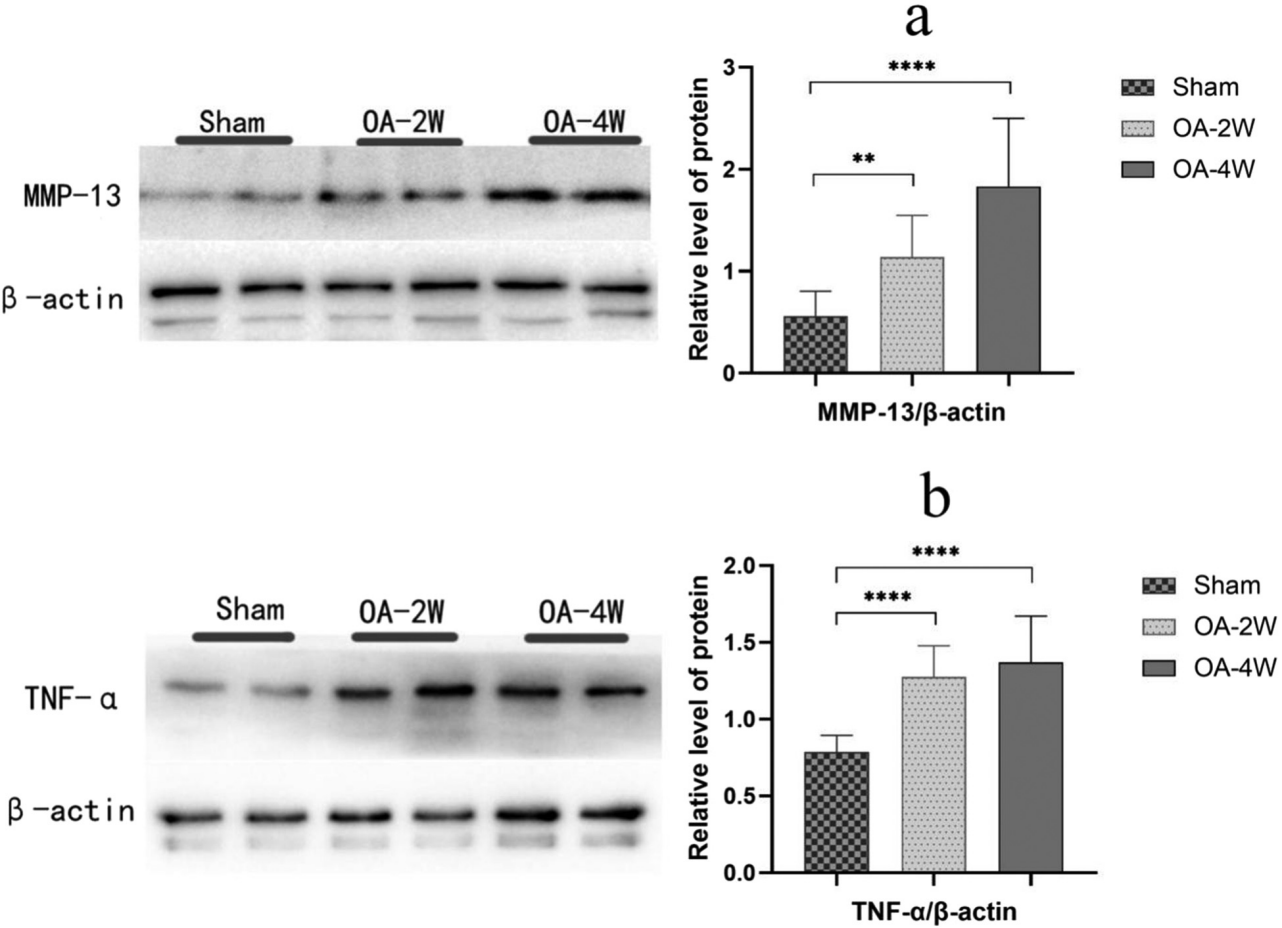
Effect of MIA treatment on cartilage inflammatory and degradative proteins. Western blot analysis of (a) MMP-13, (b) TNF-α in the distal femur containing cartilage and subchondral region is represented after treatment in rat articular. All values are expressed as Mean ± SD. (*n* = 6/group). **P < 0.01, ****P < 0.0001 compared to the sham group. TNF-α, MMP-13. Sham: animals received sterile saline 0.9% for 4 weeks; OA-2W: animals received MIA for 2 weeks; OA-4W: animals received MIA for 4 weeks.

Quantitative real-time PCR and Western blot semi-quantitative analysis was used to detect the expression of proinflammatory and cartilage-associated proteins. The progressive destruction of articular cartilage remarkably enhanced the expression of IL-1β, TNF-α, and MMP-13 and downregulated the expression of cartilage-specific protein collagen II (Figure 5). When we administered MIA for a long time, we observed aggravation of the deleterious effects of MIA. MIA increased the expression of IL-1β by approximately 7 folds in MIA two-week group, 16 folds in MIA four-week group (Figure 5a), and upregulated the expression of TNF-α by approximately 6 folds in MIA two-week group, 16 folds in MIA four-week group (Figure 5b) compared to the sham group. The expression of MMP-13 also increased with the prolonging of modeling time (Figure 5c). Along with these deleterious effects, MIA inhibited cartilage repair by diminishing extracellular matrix (ECM) component synthesis like collagen II by approximately 70% in MIA two-week group, 87% in MIA four-week group (Figure 5d) compared to that in the sham group. These differences in expression were also verified by Western blot semi-quantitative analysis (Figure 6). The expression of TNF-α and MMP-13 increased gradually with the increase of modeling time, which was significantly different compared with the sham group (Figure 6).

### 3.5 Immunofluorescence

**Figure A1:**
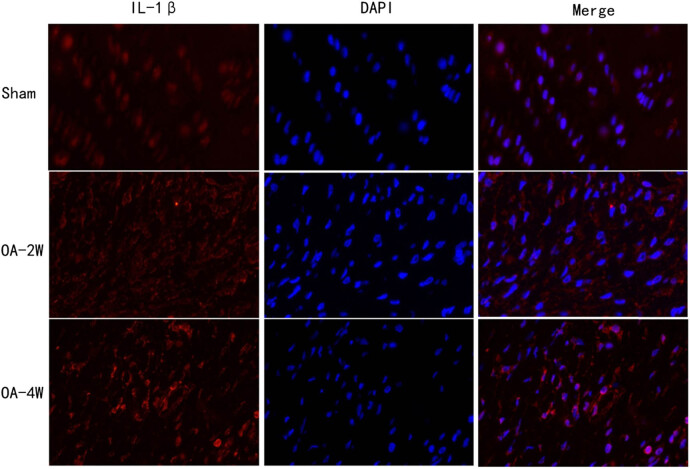
Effects of MIA treatment on the expression of IL-1β in the tibial plateau. Vision: 400× magnification. IL-1β of the tibial plateau was sparsely expressed in the sham group. The expression of IL-1β was improved by MIA treatment with the prolonging of modeling time. Sham: animals received sterile saline 0.9% for 4 weeks; OA-2W: animals received MIA for 2 weeks; OA-4W: animals received MIA for 4 weeks.

As indicated by the immunofluorescence images (Figure A1), IL-1β of the tibial plateau was sparsely expressed in the sham group. However, compared with the saline injection joints, the expression of IL-1β was significantly improved by the MIA treatment. With the prolonging of modeling time, the expression level increased gradually.

## 4 Discussion

OA is a degenerative joint disease that leads to functional disability and progressive loss of articular cartilage induced by diverse factors. In this research, we inspected the impact of OA on knee cartilage and subchondral bone in rodent models. OA in rats was induced by MIA, a widely used toxic modeling reagent, which gave rise to apoptosis of chondrocytes, subchondral bone changes, cartilage erosion, osteophytes, and cartilage clefts [19].

OA affects the subchondral bone due to the aberrant mechanical loading, which will cause microfractures in the subchondral bone and the associated increased bone resorption remodeling activity [21]. Increased osteoclastic activity and bone resorption are often observed in the subchondral bone during the early stages of OA. At later stages, the bone remodeling activity tends to bone formation, leading to abnormal bone formation and osteophytes [22,23]. It has been confirmed that microstructural changes in the subchondral bone environment are relevant to OA-related cartilage degeneration through cartilage–bone crosstalk [24]. Subchondral bone exerts important shock-absorbing functions for the overlying articular cartilage, distributes forces and adapts to maintain joint conformation and prevent stress concentration, attenuating approximately 30% of the joint load during movement [25], and experiences a constant adaptation in response to changes in the mechanical environment through modeling or remodeling [26]. Therefore, the overall properties of the subchondral bone were evaluated in this study.

Traumatic OA modeling by anterior cruciate ligament excision was found a decrease in Tb.Th and trabecular bone number at the early stage, along with an increase in bone resorption, resulting in a diminution in BMD. Although there was an increased bone formation in the subchondral bone and osteophyte in the late stage, loss of cancellous bone and thinning of bone trabeculae are observed [27]. And again, in the inflammatory OA model, the subchondral bone plate was found to be thinner as time went by, with increased porosity, trabecular loss, and reduced bone mass [28]. In parallel with these discoveries, our findings demonstrated that the Tb. Sp was higher in the MIA groups relative to the sham group. BMD, connectivity density, Tb.Th, and bone volume fraction were also observed with a corresponding decrease. These findings suggested increased bone resorption activity associated with OA.

Previous studies support that cartilage and subchondral bone communicate with each other through biomechanical pathways to maintain the homeostasis of the joint environment [29]. In this research, histopathological examination showed that MIA-induced remarkable lesions in OA, including chondrocyte degeneration, collapse and fragmentation of subchondral bone, loss of chondrocytes, and a focally extensive defect in the cartilage, which was validated by safranin-o and toluidine blue staining. MIA caused extensive damage to articular cartilage, consistent with previous findings [1,16]. After MIA injection, a strong inflammatory response is found histologically in the joint [30]. In addition, defective articular cartilage increases miscellaneous inflammatory cytokines and reduces chondrocyte synthesis, resulting in accelerated bone remodeling and deterioration of subchondral bone [29]. MIA injection was able to trigger degenerative changes of the joint and pathological morphologic changes in articular cartilage, as indicated by the higher scores for all histological aspects evaluated. Thereupon, the subsequent release of cartilage destruction products can give rise to localized inflammation of the joint. As a result, the self-perpetuating inflammation will cause more cartilage to break down [7,8].

In the light of these results, we sought to identify whether transformations in subchondral bone structure are associated with cartilage inflammatory response in rat chondrocytes. However, there is no consensus on the relationship between subchondral bone structure modifications and inflammatory responses in MIA-induced OA.

Previous studies have reported that intra-articular injection of MIA primarily causes severe acute inflammation and exacerbates degenerative changes in the cartilage [17]. It is known to us that inflammation is a crucial biological factor related to the pathogenesis of OA [31]. At the same time, the exact mechanism of OA remains vague. An increasing number of studies suggest that inflammatory factors play an important role in the downstream inflammatory cascade [32]. Previous research reports that inflammatory cytokines and other markers are higher in OA patients [33]. Inflammatory cytokines, for instance, IL-1β and TNF-α, are dominating inducers in the partial and systemic inflammatory processes of OA [34]. As a critical inflammatory cytokine, IL-1β is also closely associated with the pathological progression of OA [4].

To determine whether the structural breakdown of cartilage and the inflammatory reaction caused by MIA deteriorate with time, the level of IL-1β and TNF-α was quantified and compared. In this study, using our established MIA-induced rat model, the expression quantity of IL-1β and TNF-α was also found to be higher in MIA-treated rats compared to that in the sham group, comparable to the observation in previous MIA OA models.

Proinflammatory cytokines are known to intervene in the degradation of cartilage matrix proteins, such as aggrecan and collagen, by upregulating the gene expression level of MMPs [35] and chondrocytes apoptosis [36]. Additionally, many studies have demonstrated that IL-1β is co-expressed with OA-related MMPs, leading to cartilage degradation [37]. MMPs are mainly responsible for the degradation of arthritic cartilage and remodeling of ECM, of which collagen II is the main component [38].

MMP-13, also known as collagenase-3, is elevated during the early stages of OA. MMP-13 can restrain the synthesis of collagen and promote the degradation of ECM [36,39]. It is also known that MMP-13 expression is increased in the articular cartilage of human patients with OA [40]. MMP13 is associated with knee structural abnormalities as well as serum inflammatory factors [41].

Molecular analysis of qRT-PCR and Western blot revealed that the advanced subchondral bone structure destruction and chondrocyte apoptosis following MIA induction might be related to the imbalance of cartilage anabolic and inflammation-related genes. As exhibited in the diagram, the expression of MMP-13 is upregulated after injection of MIA, resulting in cartilage destruction. MMP-13 inhibits chondrocyte activity by reducing ECM components synthesis, obstructing the cartilage-specific proteins synthesis such as collagen II [29]. These results above manifest that MIA exerts an intense pro-inflammatory effect on chondrocytes.

These discoveries indicated that MIA treatment increases the inflammatory reaction and accelerates articular and subchondral bone loss. However, a limitation that confirms the effect of MIA on OA modeling is the progressively degenerative nature of the disease in humans, where extensive chondrocyte loss and cartilage degeneration in inflammatory OA are impossible to reverse or modify. In the early stages of OA after traumatic injury, chondrocytes undergo a compensatory change of hypertrophy, which acts as a compensatory mechanism to modify the ECM in response to increased load [42]. This pathological signaling starts at the site of injury and spreads throughout the articular surface leading to an almost full chondrocyte loss and complete degeneration of the articular surface [42]. Furthermore, given the structural parameters of subchondral trabecular bone seen *in vitro*, we hold the opinion that intra-articular injection of MIA can simulate subchondral bone structure changes during the acute phase of OA. As a result, inducing OA formation with MIA treatment may not have the same end-stage manifestations such as subchondral osteosclerosis that we observe when OA occurs naturally, slowly, and progressively.

## 5 Conclusion

Taken together, MIA substantially deteriorated articular cartilage as proved by irregular surfaces, thinning, and partial defects and increased OOCHAS score in MIA-injected OA rats, indicating aggravation of cartilage degeneration. In addition, the application of MIA accelerated the expression of representative inflammatory cytokines, such as IL-1β and TNF-α, in osteoarticular chondrocytes, further indicating an intense inflammatory effect of MIA. Different phases of MIA-induced OA were associated with the changes in subchondral bone microstructure and the progression of local inflammation.

## Appendix

**Table A1:** Sequence-specific primers used for cDNA amplified

Oligo name	Sequence (5′-3′)	Fragment length (bp)
TNF-α-F (forward primer)	CCAGGTTCTCTTCAAGGGACAA	80
TNF-α-R (reverse primer)	GGTATGAAATGGCAAATCGGCT	
β-actin-F (forward primer)	TGCTATGTTGCCCTAGACTTCG	240
β-actin-R (reverse primer)	GTTGGCATAGAGGTCTTTACGG	
MMP-13-F (forward primer)	ATGTGACACCTCTGAATTTTACCAG	228
MMP-13-R (reverse primer)	CATGGGCAGCAACAATAAATAAG	
Collagenase II-F (forward primer)	GAGCGGAGACTACTGGATTGATC	238
Collagenase II-R (reverse primer)	GACGTTAGCGGTGTTGGGAG	
